# Eleven High-Quality Reference Genome Sequences and 360 Draft Assemblies of Shiga Toxin-Producing Escherichia coli Isolates from Human, Food, Animal, and Environmental Sources in Canada

**DOI:** 10.1128/MRA.00625-19

**Published:** 2019-10-10

**Authors:** Shari Tyson, Christy-Lynn Peterson, Adam Olson, Shaun Tyler, Natalie Knox, Emma Griffiths, Damion Dooley, William Hsiao, Jennifer Cabral, Roger P. Johnson, Chad Laing, Victor Gannon, Tarah Lynch, Gary Van Domselaar, Fiona Brinkman, Morag Graham

**Affiliations:** a National Microbiology Laboratory, Public Health Agency of Canada, Winnipeg, Manitoba, Canada; b National Microbiology Laboratory, Public Health Agency of Canada, Guelph, Ontario, Canada; c National Microbiology Laboratory, Public Health Agency of Canada, Lethbridge, Alberta, Canada; d Department of Medical Microbiology and Infectious Diseases, University of Manitoba, Winnipeg, Manitoba, Canada; e Department of Molecular Biology and Biochemistry, Simon Fraser University, Vancouver, British Columbia, Canada; f British Columbia Public Health Microbiology and Reference Laboratory, Vancouver, British Columbia, Canada; University of Maryland School of Medicine

## Abstract

We report high-quality closed reference genomes for 1 bovine strain and 10 human Shiga toxin (Stx)-producing Escherichia coli (STEC) strains from serogroups O26, O45, O91, O103, O104, O111, O113, O121, O145, and O157. We also report draft assemblies, with standardized metadata, for 360 STEC strains isolated from watersheds, animals, farms, food, and human infections.

## ANNOUNCEMENT

Shiga toxin (Stx)-producing Escherichia coli (STEC) strains cause significant human enteric disease (1–3). Among >129 O serogroups, O157, O26, O45, O111, O103, O121, and O145 cause most infections (4–6). Non-O157 STEC strains are increasingly reported (5–7), with recent widespread STEC O121 and O103 outbreaks in Canada and the United States sourced to flour (8) and ground beef, respectively (https://www.cdc.gov/ecoli/2019/o103-04-19/).

To catalogue pangenomic diversity, we generated closed reference genomes for 11 routinely used lab control strains from the “top seven” STEC O serogroups, plus O91 and O104, and 360 draft assemblies for 129 distinct O serogroups from STEC culture collections (1980 to 2013) originating from watersheds, farms or foods (*n =* 238), human infections (*n =* 74), proficiency panels (*n =* 27), and unknown sources (*n =* 32). Prior to selection, isolates were traditionally serotyped at national or provincial reference labs, and *stx* gene presence/subtype was assessed by preestablished generic and differentiating *stx* PCR assays (9–11).

DNA extracted from 1-ml Luria-Bertani broth cultures grown overnight at 37°C using MasterPure complete DNA purification kits (Epicentre Technologies Corp., Chicago, IL, USA) was fragmented by an E210 ultrasonicator (Covaris, Inc., Woburn, MA, USA). TruSeq DNA library preparation v2 kit (Illumina, San Diego, CA, USA) libraries were shotgun sequenced using an Illumina GAIIx system (2 × 150-bp paired-end cluster generation kit v4 and TruSeq SBS kit v5) or MiSeq platform (2 × 300 bp; v3 chemistry). Illumina reads were managed in the Integrated Rapid Infectious Disease Analysis (IRIDA) platform (12), assessed for quality (Q > 30) using FastQC (13), and trimmed using Trimmomatic v0.34 (14). Overlapping reads merged with FLASH v1.2.11 (15) were *de novo* assembled using SPAdes v3.8.2 (16)/Shovill 0.9.0 (17). Postassembly quality control was achieved using QUAST v5.0.0 (number of contigs, < 500; reference coverage, 70% [closest polished genome of FWSEC0001-0011]) (18). Reference strains were augmented with 2 × 300-bp MiSeq (v3 chemistry) reads from mate pair (∼8 kb) TruSeq libraries and with MinION Mk1b long reads from the rapid barcoding sequencing kit (SQK-RBK004; Oxford Nanopore Technologies Ltd., Oxford, UK) libraries. Albacore v2.3.0 base-called/quality-filtered long reads were *de novo* assembled using Canu v1.7 (19) and with quality-controlled Illumina mate pair reads as hybrid assemblies using Unicycler v0.4.4.0 (20). When assemblies appeared congruent in Mauve v20150226Build10 (21), Unicycler assemblies were used. Otherwise, mate pair reads were mapped to both assemblies using Bowtie 2 v2.3.4.1 (22) and BAM files assessed for coverage/connections using GAP5 v.1.2.14-r (23); long reads were mapped with BWA-MEM v0.7.17.1 (24) and assessed using Tablet v1.17.08.17 (25). Canu contigs were employed to scaffold/correct Unicycler contigs using the Staden package GAP4 (26, 27); all contigs were circularized and trimmed. Assemblies were Illumina read polished (5 rounds) using Bowtie 2/Pilon v1.20.1 (28). NCBI’s default Web BLASTN (29) identified plasmid contigs and confirmed that *in silico* O-serogroup determinations were congruent with traditional lab determinations. Read depth was assessed using SAMtools idxstats (30). After functional annotation using NCBI’s Prokaryotic Genome Annotation Pipeline (31), assemblies were reoriented to replication origin (*dnaA*) using Circlator v1.1.5 (32).

Illumina reference genome coverage ranged from 65.6× to 130.7× (average, 96.1×); MinION coverage ranged from 51.2× to 325.1× (average, 149.6×) (Table 1). Of 360 draft assemblies, 357 yielded scaffolds (average contigs, 153.0; average coverage depth, 111.7×). Eleven reference chromosomes and all plasmids but one were circularized (0 to 3 plasmids per strain). The reference chromosomes (4,955,402 to 5,697,154 bp) contained 4,967 to 5,833 coding sequences (CDS), 22 rRNAs, 90 to 103 tRNAs, and 8 to 11 noncoding RNAs (ncRNAs), as well as bacteriophages. These genomic resources augment available data and are ideal for pathogenomics applications and machine learning.

**TABLE 1:** Characteristics and accession numbers for 11 high-quality STEC reference genomes and 360 STEC draft genome assemblies from 129 distinct serogroups sequenced for this study

Isolate identifier from this study	Traditional serogroup (*in silico* prediction)^*a*^	*stx* subtype (*in silico* prediction)^*b*^	Source	Yr of isolation	Country of isolation	SRA accession no.	WGS accession no.^*c*^	Assembly level	No. of contigs postassembly, chromosome and/or plasmid(s), size (bp) (GenBank accession no.)	No. of CDS^*d*^	Coverage (×) (technology)	Genome size (all contigs) (bp)
FWSEC0001	O26:H11	*stx*_1a_, *stx*_2a_	Clinical, human	2002	Canada		GCF_005037725	Complete genome	4, chromosome, 5,697,154 (CP031922); plasmids, 95,298 (CP031923), 6,715 (CP031924), 4,148 (CP031925)	5,833	66.48 (Illumina), 325.05 (MinION)	5,803,315
FWSEC0002	O145:NM (O145:H28)	*stx*_1a_	Clinical, human	2003	Canada		GCA_005037815	Complete genome	3, chromosome, 5,695,528 (CP031919); plasmids, 92,498 (CP031920), 2,031 (CP031921)	5,594	78.00 (Illumina), 124.40 (MinION)	5,790,057
FWSEC0003	O45:H2	*stx*_1a_	Clinical, human	2005	Canada		GCA_005037845	Complete genome	3, chromosome, 5,532,455 (CP031916); plasmids, 95,228 (CP031917), 52,940 (CP031918)	5,612	100.74 (Illumina), 244.02 (MinION)	5,680,623
FWSEC0004	O157:H7	*stx*_1a_, *stx*_2a_	Clinical, human	1987	Canada		GCA_005037735	Complete genome	3, chromosome, 5,406,250 (CP031913); plasmids, 92,754 (CP031914), 6,675 (CP031915)	5,420	130.71 (Illumina), 172.42 (MinION)	5,505,679
FWSEC0005	O111:NM (O111:H8)	*stx*_1a_, *stx*_2a_	Clinical, human	2000	Canada		GCA_005037805	Complete genome	1, chromosome, 5,132,754 (CP031912); plasmids, none detected	4,967	108.41 (Illumina), 252.38 (MinION)	5,132,754
FWSEC0006	O121:H19	*stx*_2a_	Clinical, human	2003	Canada		GCA_005037715	Complete genome	2, chromosome, 5,398,870 (CP031910); plasmid, 80,681 (CP031911)	5,344	103.62 (Illumina), 127.66 (MinION)	5,479,551
FWSEC0007	O103:H2	*stx*_1a_	Clinical, human	2004	Canada		GCA_005037795	Complete genome	2, chromosome, 5,397,605 (CP031908); plasmid, 73,224 (CP031909)	5,447	103.12 (Illumina), 58.53 (MinION)	5,470,829
FWSEC0008	O91:H21	*stx*_2d_	Clinical, human	1992	Germany		GCA_005037775	Complete genome	2, chromosome, 4,972,544 (CP031906); plasmid, 121,036 (CP031907)	4,971	98.41 (Illumina), 82.67 (MinION)	5,093,580
FWSEC0009	O104:H4	*stx*_2a_	Clinical, human	2011	Germany		GCA_005014075	Complete genome	4, chromosome, 5,277,234 (CP031902); plasmids, 88,545 (CP031903), 75,669 (CP031904), 1,549 (CP031905)	5,329	65.55 (Illumina), 110.27 (MinION)	5,442,997
FWSEC0010	O113:H21	*stx*_2a_	Clinical, human	1991	Canada		GCA_005014055	Complete genome	4, chromosome, 4,955,402 (CP031898); plasmids, 160,712 (CP031899), 7,769 (CP031900), 7,117 (CP031901)	5,006	94.45 (Illumina), 51.16 (MinION)	5,131,000
FWSEC0011	O113:H21	*stx*_2d_	Domesticated livestock, bovine, animal manure, agricultural (farm)	2004	Canada		GCA_005171095	Chromosome	6, chromosome, 5,064,577 (CP031892); plasmids, 134,626 (CP031893), 115,288 in 4 scaffolds,^*e*^ (66,896 [CP031894], 26,567 [CP031895], 12,383 [CP031896], 9,442 [CP031897])	5,173	108.45 (Illumina), 96.89 (MinION)	5,314,491
FWSEC0021	O26:H11	*stx*_1a_	Clinical, human	1980	Canada	SRR7947278	RRCV00000000	Scaffold	208		92 (Illumina)	5,330,412
FWSEC0022	O26:H11	*stx*_1a_	Domesticated livestock, bovine, animal manure, agricultural (farm)	1996	Canada	SRR7947279	RRCW00000000	Scaffold	240		89 (Illumina)	5,481,594
FWSEC0023	O26:H11	*stx*_1a_	Clinical, human	1999	Switzerland	SRR7947276	RRCX00000000	Scaffold	223		130 (Illumina)	5,419,123
FWSEC0024	O26:H11	*stx*_1a_	Clinical, human	1999	Canada	SRR7947277	RRCY00000000	Scaffold	221		82 (Illumina)	5,315,394
FWSEC0025	O103:H2	*stx*_1a_	Ground beef, solid food, bovine, buildings (grocery/retail/food store)	1997	Canada	SRR7947274	RRCZ00000000	Scaffold	181		58 (Illumina)	5,214,405
FWSEC0026	O103:H2	*stx*_1a_	Clinical, human	1995	Canada	SRR7947275	RRDA00000000	Scaffold	198		56 (Illumina)	5,135,561
FWSEC0027	O103:H2	*stx*_1a_	Domesticated livestock, bovine, animal manure, agricultural (farm)	2001	Canada	SRR7947272	RRDB00000000	Scaffold	220		83 (Illumina)	5,413,960
FWSEC0028	O103:H2	*stx*_1a_, *stx*_2a_	Clinical, human	2001	Switzerland	SRR7947273	RRDC00000000	Scaffold	159		82 (Illumina)	5,107,141
FWSEC0029	O45:H2	*stx*_1a_	Domesticated livestock, bovine, animal manure, agricultural (farm)	1994	Canada	SRR7947280	RRDD00000000	Scaffold	184		215 (Illumina)	5,378,562
FWSEC0030	O45:H2	*stx*_1a_	Clinical, human	1995	USA	SRR7947281	RRDE00000000	Scaffold	173		222 (Illumina)	5,134,981
FWSEC0031	O45:H2	*stx*_1a_	Clinical, human	1996	USA	SRR7947308	RRDF00000000	Scaffold	193		213 (Illumina)	5,496,950
FWSEC0032	O45:H2	*stx*_1a_	Clinical, human	1989	Canada	SRR7947309	RRDG00000000	Scaffold	165		199 (Illumina)	5,158,875
FWSEC0033	O111:H8	*stx*_1a_	Domesticated livestock, bovine, animal manure, agricultural (farm)	1993	Canada	SRR7947306	RRDH00000000	Scaffold	214		194 (Illumina)	5,281,723
FWSEC0034	O111:H8	*stx*_1a_	Domesticated livestock, bovine, animal manure, agricultural (farm)	2000	Canada	SRR7947307	RRDI00000000	Scaffold	209		177 (Illumina)	5,270,854
FWSEC0035	O111:NM	*stx*_1a_	Clinical, human	2000	Switzerland	SRR7947304	RRDJ00000000	Scaffold	179		212 (Illumina)	5,159,316
FWSEC0036	O111:NM	*stx*_1a_	Domesticated livestock, bovine, animal manure, agricultural (farm)	2002	Canada	SRR7947305	RRDK00000000	Scaffold	187		222 (Illumina)	5,281,033
FWSEC0039	O121:H19	*stx*_2a_	Clinical, human	2002	Switzerland	SRR7947302	RRDL00000000	Scaffold	127		196 (Illumina)	5,130,319
FWSEC0040	O121:H19	*stx*_2a_	Domesticated livestock, bovine, animal manure, agricultural (farm)	2004	Canada	SRR7947303	RRDM00000000	Scaffold	150		221 (Illumina)	5,182,763
FWSEC0041	O145:NM (O145:H28)	*stx*_1a_	Clinical, human	1997	Switzerland	SRR7947300	RRDN00000000	Scaffold	192		196 (Illumina)	5,358,040
FWSEC0042	O145:H25	*stx*_2a_	Clinical, human	1998	Switzerland	SRR7947301	RRDO00000000	Scaffold	174		189 (Illumina)	5,245,327
FWSEC0043	O145:NM (O145:H28)	*stx*_2a_	Clinical, human	1999	Argentina	SRR7947248	RRDP00000000	Scaffold	169		190 (Illumina)	5,178,179
FWSEC0044	O145:NM (O145:H28)	*stx*_1a_, *stx*_2a_	Clinical, human	2001	Switzerland	SRR7947247	RRDQ00000000	Scaffold	162		177 (Illumina)	5,208,478
FWSEC0045	O91:NM (O91:H14)	*stx*_1a_, *stx*_2b_	Domesticated livestock, bovine, animal manure, agricultural (farm)	1994	USA	SRR7947246	RRDR00000000	Scaffold	252		186 (Illumina)	5,459,036
FWSEC0046	O91:NM (O91:H14)	*stx*_1a_, *stx*_2b_	Domesticated livestock, bovine, animal manure, agricultural (farm)	1994	USA	SRR7947245	RRDS00000000	Scaffold	251		191 (Illumina)	5,528,103
FWSEC0047	O91:H21	*stx*_2d_	Clinical, human	Missing	USA	SRR7947244	RRDT00000000	Scaffold	109		210 (Illumina)	4,899,053
FWSEC0048	O91:H21	*stx*_1a_, *stx*_2a_	Domesticated livestock, bovine, animal manure, agricultural (farm)	2001	Canada	SRR7947243	RRDU00000000	Scaffold	133		165 (Illumina)	5,100,887
FWSEC0049	O113:H4	*stx*_1a_, *stx*_2d_	Domesticated livestock, bovine, animal manure, agricultural (farm)	1996	Canada	SRR7947242	RRDV00000000	Scaffold	170		226 (Illumina)	4,987,637
FWSEC0050	O113:H4	*stx*_1a_, *stx*_2d_	Domesticated livestock, bovine, animal manure, agricultural (farm)	1996	Canada	SRR7947241	RRDW00000000	Scaffold	196		139 (Illumina)	4,951,257
FWSEC0051	O113:H21	*stx*_2d_	Clinical, human	1989	Canada	SRR7947250	RRDX00000000	Scaffold	122		196 (Illumina)	5,251,997
FWSEC0052	O128:NM (O128:H2)	*stx*_1c_	Clinical, human	1996	Germany	SRR7947249	RRDY00000000	Scaffold	196		196 (Illumina)	5,398,882
FWSEC0053	O128:H2	*stx*_1c_	Clinical, human	1988	Switzerland	SRR7947252	RRDZ00000000	Scaffold	213		240 (Illumina)	5,388,481
FWSEC0054	O128:H10	*stx*_1_ (partial); *stx*_1a_, *stx*_1b_ (SRST2), *stx*_1a_ (KAT)	Clinical, human	1999	Switzerland	SRR7947253	RREA00000000	Scaffold	141		187 (Illumina)	4,942,536
FWSEC0055	O113:H21	*stx*_2a_	Domesticated livestock, bovine, animal manure, agricultural (farm)	2000	Canada	SRR7947254	RREB00000000	Scaffold	120		199 (Illumina)	4,949,372
FWSEC0057	O185:H28	*stx*_1a_, *stx*_2a_	River water	2012	Canada	SRR7947255	RREC00000000	Scaffold	419		46 (Illumina)	4,862,678
FWSEC0058	O153:NM (O153:H2)	*stx*_2f_	River water	2012	Canada	SRR7947256	RRED00000000	Scaffold	500		52 (Illumina)	5,232,124
FWSEC0062	O182/O109:NM (O109:H16)	*stx*_2a_, *stx*_2g_	River water	2012	Canada	SRR7947260	RREH00000000	Scaffold	158		154 (Illumina)	5,214,988
FWSEC0063	O182/O109:NM (O109:H16)	*stx*_2a_, *stx*_2g_	River water	2012	Canada	SRR7947261	RREI00000000	Scaffold	195		127 (Illumina)	5,205,797
FWSEC0064	O88:H25	*stx*_1a_, *stx*_2a_	Stream water	2012	Canada	SRR7947373	RREJ00000000	Scaffold	160		77 (Illumina)	4,836,164
FWSEC0066	O8:H19	*stx*_2c_	River water	2012	Canada	SRR7947375	RREL00000000	Scaffold	135		72 (Illumina)	5,198,072
FWSEC0067	O8:H19	*stx*_2c_	River water	2012	Canada	SRR7947374	RREM00000000	Scaffold	144		99 (Illumina)	5,172,338
FWSEC0069	O84:NM (O84:H2)	*stx*_1a_	Municipal drain water	2012	Canada	SRR7947376	RREO00000000	Scaffold	194		185 (Illumina)	5,368,100
FWSEC0070	O121:NM (O121:H19)	*stx*_2a_	Solid stool, clinical, human	1999	Canada	SRR7947379	RREP00000000	Scaffold	133		129 (Illumina)	5,000,333
FWSEC0071	O156:NM (O156:H25)	*stx*_1a_	Solid stool, clinical, human	2004	Canada	SRR7947378	RREQ00000000	Scaffold	160		64 (Illumina)	5,054,573
FWSEC0072	O26:H11	*stx*_1a_	Proficiency panel isolate, human	2005	Denmark	SRR7947381	RRER00000000	Scaffold	205		97 (Illumina)	5,362,334
FWSEC0073	O145:H28	*stx*_1a_	Proficiency panel isolate, human	2005	Denmark	SRR7947380	RRES00000000	Scaffold	142		103 (Illumina)	5,343,649
FWSEC0074	O75:H8	*stx*_1c_, *stx*_2b_	Clinical, human	2005	Canada	SRR7947223	RRET00000000	Scaffold	153		79 (Illumina)	5,748,156
FWSEC0075	O26:H11	*stx*_1a_	Solid stool, clinical, human	2005	Canada	SRR7947224	RREU00000000	Scaffold	239		81 (Illumina)	5,396,470
FWSEC0076	O1:H20 (O1:H21)	*stx*_2a_	Clinical, human	2006	Canada	SRR7947221	RREV00000000	Scaffold	81		92 (Illumina)	5,009,613
FWSEC0077	O38:H26	*stx*_1c_, *stx*_2b_	Proficiency panel isolate, human	2006	Denmark	SRR7947222	RREW00000000	Scaffold	108		69 (Illumina)	5,138,266
FWSEC0078	O51:H49	*stx*_2e_	Proficiency panel isolate, human	2006	Denmark	SRR7947227	RREX00000000	Scaffold	201		102 (Illumina)	5,378,358
FWSEC0079	O55:H7	*stx*_2d_	Solid stool, clinical, human	2006	Canada	SRR7947228	RREY00000000	Scaffold	125		90 (Illumina)	5,285,787
FWSEC0080	O119:H25 (O182:H25)	*stx*_1a_	Food	2006	Canada	SRR7947225	RREZ00000000	Scaffold	156		90 (Illumina)	5,146,192
FWSEC0081	O NT:NM (O177:H25)	*stx*_2c_ (partial); *stx*_2d_ (SRST2); *stx*_2c_, *stx*_2d_ (KAT)	Solid stool, clinical, human	2006	Canada	SRR7947226	RRFA00000000	Scaffold	185		136 (Illumina)	5,008,627
FWSEC0082	O21:H8	*stx*_2a_	Proficiency panel isolate	2007	Denmark	SRR7947229	RRFB00000000	Scaffold	188		53 (Illumina)	4,960,003
FWSEC0083	O28ab:NM (O28:H9)	*stx*_2a_	Proficiency panel isolate	2007	Denmark	SRR7947230	RRFC00000000	Scaffold	142		99 (Illumina)	4,996,535
FWSEC0084	O26:H11	*stx*_1a_	Solid stool, clinical, human	2007	Canada	SRR7947334	RRFD00000000	Scaffold	229		106 (Illumina)	5,294,352
FWSEC0086	O110:H28	*stx*_2a_	Unknown specimen type	2009	USA	SRR7947332	RRFF00000000	Scaffold	107		74 (Illumina)	4,944,331
FWSEC0087	O104:H7 (O104:H19)	*stx*_2b_	Unknown specimen type	2009	USA	SRR7947331	RRFG00000000	Scaffold	237		128 (Illumina)	5,466,655
FWSEC0088	O76:H19	*stx*_1c_, *stx*_2b_	Proficiency panel isolate	2009	Denmark	SRR7947338	RRFH00000000	Scaffold	138		113 (Illumina)	5,327,960
FWSEC0089	O113:H4	*stx*_1c_, *stx*_2b_	Proficiency panel isolate	2009	Denmark	SRR7947337	RRFI00000000	Scaffold	134		71 (Illumina)	5,110,727
FWSEC0091	O128ab:H2 (O128:H2)	*stx*_1c_, *stx*_2b_	Proficiency panel isolate	2009	Denmark	SRR7947335	RRFJ00000000	Scaffold	195		89 (Illumina)	5,567,620
FWSEC0092	O91:H14	*stx*_2b_	Proficiency panel isolate	2009	Denmark	SRR7947330	RRFK00000000	Scaffold	101		87 (Illumina)	5,105,837
FWSEC0093	O146:H28	*stx*_2b_	Proficiency panel isolate	2009	Denmark	SRR7947329	RRFL00000000	Scaffold	159		127 (Illumina)	5,363,081
FWSEC0094	O26:H11	*stx*_1a_, *stx*_2a_	Solid stool, clinical, human	2009	Canada	SRR7947355	RRFM00000000	Scaffold	209		113 (Illumina)	5,482,763
FWSEC0095	O116:H NT (O116:H21)	*stx*_1a_, *stx*_2a_	Unknown specimen type	2009	USA	SRR7947356	RRFN00000000	Scaffold	137		107 (Illumina)	5,147,136
FWSEC0096	O8:H19	*stx*_1a_, *stx*_2d_	Unknown specimen type	2009	USA	SRR7947357	RRFO00000000	Scaffold	81		93 (Illumina)	4,890,473
FWSEC0097	O130:H11	*stx*_2a_	Unknown specimen type	2009	USA	SRR7947358	RRFP00000000	Scaffold	90		81 (Illumina)	5,054,940
FWSEC0098	O6:H34	*stx*_2a_	Unknown specimen type	2009	USA	SRR7947351	RRFQ00000000	Scaffold	93		77 (Illumina)	5,170,195
FWSEC0100	O26:H11	*stx*_1a_	Solid stool, clinical, human	2009	Canada	SRR7947353	RRFR00000000	Scaffold	201		156 (Illumina)	5,402,804
FWSEC0101	O8:H16	*stx*_1a_, *stx*_2a_	Solid stool, clinical, human	2009	Canada	SRR7947354	RRFS00000000	Scaffold	70		133 (Illumina)	4,891,263
FWSEC0102	O98:H29 (O98:H21)	*stx*_1a_	Clinical, human	2009	Canada	SRR7947359	RRFT00000000	Scaffold	160		147 (Illumina)	5,223,295
FWSEC0103	O165:NM (O165:H25)	*stx*_1a_, *stx*_2a_	Solid stool, clinical, human	2009	Canada	SRR7947360	RRFU00000000	Scaffold	166		141 (Illumina)	4,927,569
FWSEC0104	O26:H11	*stx*_1a_	Solid stool, clinical, human	2010	Canada	SRR7947289	RRFV00000000	Scaffold	203		138 (Illumina)	5,283,037
FWSEC0105	O156:H25	*stx*_1a_	Unknown specimen type	2010	USA	SRR7947288	RRFW00000000	Scaffold	146		82 (Illumina)	5,029,646
FWSEC0106	O165:H25	*stx*_1a_, *stx*_2a_	Unknown specimen type	2010	USA	SRR7947291	RRFX00000000	Scaffold	154		93 (Illumina)	4,919,952
FWSEC0107	O79:H7	*stx*_2c_	Unknown specimen type	2010	USA	SRR7947290	RRFY00000000	Scaffold	105		45 (Illumina)	4,882,294
FWSEC0108	O39:H49	*stx*_1a_, *stx*_2a_	Unknown specimen type	2010	USA	SRR7947285	RRFZ00000000	Scaffold	101		88 (Illumina)	5,008,177
FWSEC0109	O26:NM (O26:H11)	*stx*_1a_	Unknown specimen type	2010	Canada	SRR7947284	RRGA00000000	Scaffold	241		94 (Illumina)	5,339,476
FWSEC0110	O103:H2	*stx*_1a_	Clinical, human	2010	Canada	SRR7947340	RRGB00000000	Scaffold	192		131 (Illumina)	5,341,967
FWSEC0111	O174:H8	*stx*_1c_, *stx*_2b_	Proficiency panel isolate	2010	Denmark	SRR7947339	RRGC00000000	Scaffold	115		109 (Illumina)	5,029,489
FWSEC0112	O41:H26	*stx*_1d_	Proficiency panel isolate	2010	Denmark	SRR7947361	RRGD00000000	Scaffold	120		117 (Illumina)	5,443,975
FWSEC0113	O6:H2 (O103:H2)	*stx*_1a_	Unknown specimen type	2010	Canada	SRR7947328	RRGE00000000	Scaffold	184		72 (Illumina)	5,255,259
FWSEC0114	O139:H1	*stx*_2e_	Proficiency panel isolate	Missing	Denmark	SRR7947318	RRGF00000000	Scaffold	135		76 (Illumina)	5,146,693
FWSEC0115	O171:H2	*stx*_2c_ (partial), *stx*_2_ (SRST2), *stx*_2d_ (KAT)	Proficiency panel isolate	Missing	Denmark	SRR7947319	RRGG00000000	Scaffold	151		79 (Illumina)	5,161,156
FWSEC0116	O91:H21	*stx*_2d_	Proficiency panel isolate	Missing	Denmark	SRR7947316	RRGH00000000	Scaffold	97		86 (Illumina)	4,929,810
FWSEC0117	O145:H34	*stx*_2f_	Proficiency panel isolate	Missing	Denmark	SRR7947317	RRGI00000000	Scaffold	105		65 (Illumina)	4,910,022
FWSEC0118	O2:H25	*stx*_2g_	Proficiency panel isolate	Missing	Denmark	SRR7947314	RRGJ00000000	Scaffold	135		83 (Illumina)	5,348,740
FWSEC0119	O146:H21	*stx*_1c_, *stx*_2a_	Proficiency panel isolate	Missing	Denmark	SRR7947315	RRGK00000000	Scaffold	186		97 (Illumina)	5,423,256
FWSEC0120	O154:H31	*stx*_1d_	Proficiency panel isolate	Missing	Denmark	SRR7947282	RRGL00000000	Scaffold	81		73 (Illumina)	5,305,349
FWSEC0121	O22:H8	*stx*_1c_, *stx*_2b_	Proficiency panel isolate	Missing	Denmark	SRR7947283	RRGM00000000	Scaffold	193		87 (Illumina)	5,397,345
FWSEC0122	O48:H21	*stx*_1a_, *stx*_2a_	Proficiency panel isolate	Missing	Denmark	SRR7947310	RRGN00000000	Scaffold	105		99 (Illumina)	5,036,092
FWSEC0123	O174:H21	*stx*_2c_	Proficiency panel isolate	Missing	Denmark	SRR7947311	RRGO00000000	Scaffold	101		161 (Illumina)	5,239,138
FWSEC0124	O118:H12	*stx*_2b_	Proficiency panel isolate	Missing	Denmark	SRR7947236	RRGP00000000	Scaffold	146		92 (Illumina)	5,011,013
FWSEC0125	O73:H18 (O17:H18)	*stx*_2d_	Proficiency panel isolate	Missing	Denmark	SRR7947235	RRGQ00000000	Scaffold	72		82 (Illumina)	5,126,562
FWSEC0126	O8:K85ab:H rough (O8:H19)	*stx*_1d_	Proficiency panel isolate	Missing	Denmark	SRR7947238	RRGR00000000	Scaffold	77		108 (Illumina)	5,592,160
FWSEC0127	O128ab:NM (O128:H2)	*stx*_2f_	Proficiency panel isolate	Missing	Denmark	SRR7947237	RRGS00000000	Scaffold	164		80 (Illumina)	5,355,188
FWSEC0128	O183:H21 (O183:H18)	*stx*_1a_, *stx*_2a_	Solid stool, clinical, human	2010	Canada	SRR7947232	RRGT00000000	Scaffold	85		72 (Illumina)	5,293,073
FWSEC0129	O71:H11	*stx*_1a_	Solid stool, clinical, human	2011	Canada	SRR7947231	RRGU00000000	Scaffold	215		70 (Illumina)	5,212,364
FWSEC0131	O118:H16	*stx*_1a_	Solid stool, clinical, human	2011	Canada	SRR7947233	RRGW00000000	Scaffold	216		112 (Illumina)	5,431,187
FWSEC0132	O177:NM (O177:H25)	*stx*_2c_	Solid stool, clinical, human	2011	Canada	SRR7947240	RRGX00000000	Scaffold	204		86 (Illumina)	4,960,664
FWSEC0133	O26:H11	*stx*_1a_	Solid stool, clinical, human	2011	Canada	SRR7947239	RRGY00000000	Scaffold	229		102 (Illumina)	5,308,084
FWSEC0135	O49:NM (O49:H16)	*stx*_2a_	Solid stool, clinical, human	2011	Canada	SRR7947389	RRHA00000000	Scaffold	186		102 (Illumina)	4,775,607
FWSEC0136	O103:H21 (O103:H2)	*stx*_1a_	Solid stool, clinical, human	2011	Canada	SRR7947386	RRHB00000000	Scaffold	172		83 (Illumina)	5,325,986
FWSEC0137	O103:H2 (O103:H25)	*stx*_1a_	Solid stool, clinical, human	2011	Canada	SRR7947387	RRHC00000000	Scaffold	173		78 (Illumina)	5,127,832
FWSEC0138	O103:H2	*stx*_1a_	Solid stool, clinical, human	2011	Canada	SRR7947384	RRHD00000000	Scaffold	157		69 (Illumina)	5,264,166
FWSEC0139	O26:H21 (O26:H11)	*stx*_1a_	Solid stool, clinical, human	2011	Canada	SRR7947385	RRHE00000000	Scaffold	225		65 (Illumina)	5,449,882
FWSEC0140	O121:H1 (O121:H19)	*stx*_2a_	Solid stool, clinical, human	2011	Canada	SRR7947382	RRHF00000000	Scaffold	143		71 (Illumina)	5,127,687
FWSEC0141	O69:H11	*stx*_1a_	Solid stool, clinical, human	2010	Canada	SRR7947383	RRHG00000000	Scaffold	214		96 (Illumina)	5,495,125
FWSEC0142	O26:H21 (O26:H11)	*stx*_1a_	Solid stool, clinical, human	2011	Canada	SRR7947390	RRHH00000000	Scaffold	229		81 (Illumina)	5,277,599
FWSEC0143	O103:H2	*stx*_1a_	Solid stool, clinical, human	2011	Canada	SRR7947391	RRHI00000000	Scaffold	225		44 (Illumina)	5,201,971
FWSEC0144	O26:H11	*stx*_1a_	Unknown specimen type	2011	Canada	SRR7947367	RRHJ00000000	Scaffold	234		72 (Illumina)	5,303,448
FWSEC0146	O103:H25	*stx*_1a_	Clinical, human	2011	Canada	SRR7947365	RRHL00000000	Scaffold	181		149 (Illumina)	5,390,238
FWSEC0147	O103:H2	*stx*_1a_	Clinical, human	2011	Canada	SRR7947364	RRHM00000000	Scaffold	161		113 (Illumina)	5,304,488
FWSEC0150	O1:H rough (O1:H20)	*stx*_2a_	Clinical, human	2011	Canada	SRR7947369	RRHP00000000	Scaffold	83		134 (Illumina)	5,049,946
FWSEC0151	O111:NM (O111:H8)	*stx*_1a_	Solid stool, clinical, human	2001	Canada	SRR7947368	RRHQ00000000	Scaffold	189		144 (Illumina)	5,347,332
FWSEC0152	O111:NM (O111:H8)	*stx*_1a_	Clinical, human	2010	Canada	SRR7947363	RRHR00000000	Scaffold	179		118 (Illumina)	5,193,004
FWSEC0153	O121:H19	*stx*_2a_	Solid stool, clinical, human	2010	Canada	SRR7947362	RRHS00000000	Scaffold	166		156 (Illumina)	5,078,658
FWSEC0154	O121:H19	*stx*_2a_	Solid stool, clinical, human	2011	Canada	SRR7947347	RRHT00000000	Scaffold	169		145 (Illumina)	5,218,466
FWSEC0155	O121:H19	*stx*_2a_	Solid stool, clinical, human	2011	Canada	SRR7947348	RRHU00000000	Scaffold	182		112 (Illumina)	5,077,668
FWSEC0156	O121:H19	*stx*_2a_	Solid stool, clinical, human	2011	Canada	SRR7947349	RRHV00000000	Scaffold	159		178 (Illumina)	5,156,591
FWSEC0157	O111:NM (O111:H8)	*stx*_1a_	Solid stool, clinical, human	2010	Canada	SRR7947350	RRHW00000000	Scaffold	192		183 (Illumina)	5,287,703
FWSEC0158	O111:NM (O111:H8)	*stx*_1a_	Solid stool, clinical, human	2011	Canada	SRR7947343	RRHX00000000	Scaffold	196		210 (Illumina)	5,346,730
FWSEC0159	O111:NM (O111:H8)	*stx*_1a_, *stx*_2a_	Solid stool, clinical, human	2011	Canada	SRR7947344	RRHY00000000	Scaffold	244		183 (Illumina)	5,359,848
FWSEC0160	O121:H19	*stx*_2a_	Solid stool, clinical, human	2011	Canada	SRR7947345	RRHZ00000000	Scaffold	167		173 (Illumina)	5,118,519
FWSEC0161	O121:H19	*stx*_2a_	Clinical, human	2011	Canada	SRR7947346	RRIA00000000	Scaffold	161		219 (Illumina)	5,114,368
FWSEC0233	O8:H19	*stx*_2c_	River water	2012	Canada	SRR7612229	RRIB00000000	Scaffold	70		111 (Illumina)	4,959,778
FWSEC0234	O45:NM (O45:H2)	*stx*_2f_	River water	2012	Canada	SRR7612228	RRIC00000000	Scaffold	139		118 (Illumina)	5,310,469
FWSEC0235	O45:NM (O45:H2)	*stx*_2f_	River water	2012	Canada	SRR7612231	RRID00000000	Scaffold	135		116 (Illumina)	5,308,760
FWSEC0236	O45:NM (O45:H2)	*stx*_2f_	River water	2012	Canada	SRR7612230	RRIE00000000	Scaffold	133		105 (Illumina)	5,313,318
FWSEC0237	O45:NM (O45:H2)	*stx*_2f_	River water	2012	Canada	SRR7612225	RRIF00000000	Scaffold	134		87 (Illumina)	5,310,915
FWSEC0238	O45:NM (O45:H2)	*stx*_2f_	River water	2012	Canada	SRR7612224	RRIG00000000	Scaffold	141		89 (Illumina)	5,316,450
FWSEC0239	O45:NM (O45:H2)	*stx*_2f_	River water	2012	Canada	SRR7612227	RRIH00000000	Scaffold	133		102 (Illumina)	5,317,769
FWSEC0241	O45:NM (O45:H2)	*stx*_2f_	River water	2012	Canada	SRR7612233	RRIJ00000000	Scaffold	133		96 (Illumina)	5,314,331
FWSEC0242	O45:NM (O45:H2)	*stx*_2f_	River water	2012	Canada	SRR7612232	RRIK00000000	Scaffold	150		68 (Illumina)	5,311,201
FWSEC0244	O45:NM (O45:H2)	*stx*_2f_	River water	2012	Canada	SRR7612362	RRIM00000000	Scaffold	243		121 (Illumina)	5,568,249
FWSEC0245	O45:NM (O45:H2)	*stx*_2f_	River water	2012	Canada	SRR7612181	RRIN00000000	Scaffold	145		51 (Illumina)	5,309,767
FWSEC0246	O45:NM (O45:H2)	*stx*_2f_	River water	2012	Canada	SRR7612182	RRIO00000000	Scaffold	135		114 (Illumina)	5,317,036
FWSEC0247	O45:NM (O45:H2)	*stx*_2f_	River water	2012	Canada	SRR7612175	RRIP00000000	Scaffold	131		51 (Illumina)	5,309,640
FWSEC0248	O4 (O135):NM (O4:H2)	*stx*_2f_	River water	2012	Canada	SRR7612176	RRIQ00000000	Scaffold	131		125 (Illumina)	5,212,374
FWSEC0249	O45:NM (O45:H2)	*stx*_2f_	River water	2012	Canada	SRR7612177	RRIR00000000	Scaffold	132		80 (Illumina)	5,309,552
FWSEC0251	O45:NM (O45:H2)	*stx*_2f_	River water	2012	Canada	SRR7612223	RRIT00000000	Scaffold	132		72 (Illumina)	5,317,712
FWSEC0252	O45:NM (O45:H2)	*stx*_2f_	River water	2012	Canada	SRR7612304	RRIU00000000	Scaffold	144		143 (Illumina)	5,310,446
FWSEC0253	O45:NM (O45:H2)	*stx*_2f_	River water	2012	Canada	SRR7612506	RRIV00000000	Scaffold	139		79 (Illumina)	5,307,818
FWSEC0254	O45:NM (O45:H2)	*stx*_2f_	River water	2012	Canada	SRR7612505	RRIW00000000	Scaffold	135		49 (Illumina)	5,282,918
FWSEC0255	O45:NM (O45:H2)	*stx*_2f_	River water	2012	Canada	SRR7612504	RRIX00000000	Scaffold	135		88 (Illumina)	5,314,657
FWSEC0256	O91:H21	*stx*_2a_	Ground beef, solid food, bovine, buildings (grocery/retail/food store)	2010	Canada	SRR7612503	RRIY00000000	Scaffold	106		143 (Illumina)	4,939,455
FWSEC0257	O91:H14	*stx*_1a_	River water	2011	Canada	SRR7612510	RRIZ00000000	Scaffold	96		120 (Illumina)	5,291,420
FWSEC0258	O111:H8	*stx*_1a_, *stx*_2a_	River water	2011	Canada	SRR7612509	RRJA00000000	Scaffold	147		81 (Illumina)	5,104,080
FWSEC0259	O1:H20	*stx*_2a_	River water	2011	Canada	SRR7612508	RRJB00000000	Scaffold	67		122 (Illumina)	5,067,012
FWSEC0260	O111:H8	*stx*_1a_, *stx*_2a_	Ground beef, solid food, bovine, buildings (grocery/retail/food store)	2011	Canada	SRR7612507	RRJC00000000	Scaffold	150		76 (Illumina)	5,110,497
FWSEC0261	O111:NM (O111:H8)	*stx*_1a_	River water	2011	Canada	SRR7612512	RRJD00000000	Scaffold	137		107 (Illumina)	5,137,935
FWSEC0262	O91:H14	*stx*_1a_	River water	2011	Canada	SRR7612511	RRJE00000000	Scaffold	101		107 (Illumina)	5,325,101
FWSEC0263	O1:H20	*stx*_1a_	River water	2011	Canada	SRR7612467	RRJF00000000	Scaffold	72		114 (Illumina)	5,026,605
FWSEC0264	O26:H11	*stx*_1a_	River water	2011	Canada	SRR7612468	RRJG00000000	Scaffold	186		136 (Illumina)	5,305,929
FWSEC0265	O103:H2	*stx*_1a_	River water	2011	Canada	SRR7612465	RRJH00000000	Scaffold	115		85 (Illumina)	5,327,519
FWSEC0266	O1:H20	*stx*_1a_	River water	2012	Canada	SRR7612466	RRJI00000000	Scaffold	70		82 (Illumina)	5,064,420
FWSEC0267	O103:H2	*stx*_1a_	River water	2012	Canada	SRR7612471	RRJJ00000000	Scaffold	141		122 (Illumina)	5,327,547
FWSEC0268	O1:H20	*stx*_1a_	Domesticated livestock, bovine, animal manure, agricultural (farm)	2012	Canada	SRR7612472	RRJK00000000	Scaffold	49		98 (Illumina)	4,974,621
FWSEC0269	O1:H20	*stx*_1a_	Domesticated livestock, bovine, animal manure, agricultural (farm)	2012	Canada	SRR7612469	RRJL00000000	Scaffold	64		106 (Illumina)	5,021,634
FWSEC0270	O103:H2	*stx*_1a_	Water	2012	Canada	SRR7612470	RRJM00000000	Scaffold	135		67 (Illumina)	5,301,031
FWSEC0271	O103:H2	*stx*_1a_	Water	2012	Canada	SRR7612463	RRJN00000000	Scaffold	143		97 (Illumina)	5,362,142
FWSEC0272	O174:H21	*stx*_2d_	Water	2012	Canada	SRR7612464	RRJO00000000	Scaffold	143		121 (Illumina)	5,247,426
FWSEC0273	O26:H11	*stx*_1a_	Water	2012	Canada	SRR7612375	RRJP00000000	Scaffold	180		72 (Illumina)	5,246,449
FWSEC0274	O145:NM (O145:H28)	*stx*_2a_	Water	2012	Canada	SRR7612559	RRJQ00000000	Scaffold	139		86 (Illumina)	5,169,990
FWSEC0275	O26:H11	*stx*_1a_	Water	2012	Canada	SRR7612336	RRJR00000000	Scaffold	182		83 (Illumina)	5,238,710
FWSEC0276	O26:NM (O26:H11)	*stx*_1a_	Water	2012	Canada	SRR7612335	RRJS00000000	Scaffold	179		85 (Illumina)	5,384,889
FWSEC0277	O26:H11	*stx*_1a_	Water	2012	Canada	SRR7612338	RRJT00000000	Scaffold	201		81 (Illumina)	5,371,254
FWSEC0278	O104:H7	*stx*_2a_	Water	2012	Canada	SRR7612337	RRJU00000000	Scaffold	58		57 (Illumina)	4,996,585
FWSEC0279	O103:H2	*stx*_1a_	Water	2012	Canada	SRR7612340	RRJV00000000	Scaffold	128		96 (Illumina)	5,432,377
FWSEC0280	O103:H2	*stx*_1a_	Water	2013	Canada	SRR7612339	RRJW00000000	Scaffold	120		113 (Illumina)	5,200,889
FWSEC0281	O118:H16	*stx*_1a_	Water	2013	Canada	SRR7612556	RRJX00000000	Scaffold	178		81 (Illumina)	5,483,599
FWSEC0282	O91:H21	*stx*_1a_, *stx*_2d_, *stx*_2c_	Domesticated livestock, bovine, animal manure, agricultural (farm)	2013	Canada	SRR7612555	RRJY00000000	Scaffold	83		92 (Illumina)	5,033,695
FWSEC0283	O1:H20	*stx*_1a_	Domesticated livestock, bovine, animal manure, agricultural (farm)	2013	Canada	SRR7612244	RRJZ00000000	Scaffold	62		108 (Illumina)	5,089,114
FWSEC0284	O174:H21	*stx*_2a_	Ground beef, solid food, bovine, buildings (grocery/retail/food store)	2013	Canada	SRR7612245	RRKA00000000	Scaffold	82		95 (Illumina)	5,077,035
FWSEC0285	O103:H11	*stx*_1a_	Water	2013	Canada	SRR7612089	RRKB00000000	Scaffold	193		81 (Illumina)	5,304,443
FWSEC0286	O103:H25	*stx*_1a_	Domesticated livestock, bovine, animal manure, agricultural (farm)	2013	Canada	SRR7612095	RRKC00000000	Scaffold	154		98 (Illumina)	5,289,604
FWSEC0287	O103:H25	*stx*_1a_	Domesticated livestock, bovine, animal manure, agricultural (farm)	2013	Canada	SRR7612248	RRKD00000000	Scaffold	156		76 (Illumina)	5,276,158
FWSEC0289	O153:H25	*stx*_1a_, *stx*_2a_	Domesticated livestock, bovine, animal manure, agricultural (farm)	2013	Canada	SRR7612250	RRKE00000000	Scaffold	90		67 (Illumina)	4,909,247
FWSEC0290	O113:H21	*stx*_2a_ (partial), *stx*_2a_ (SRST2), *stx*_2a_ (KAT)	Domesticated livestock, bovine, animal manure, agricultural (farm)	2013	Canada	SRR7612251	RRKF00000000	Scaffold	112		97 (Illumina)	5,070,644
FWSEC0291	O113:H21	*stx*_1a_, *stx*_2a_	Domesticated livestock, bovine, animal manure, agricultural (farm)	2013	Canada	SRR7612105	RRKG00000000	Scaffold	86		99 (Illumina)	5,165,323
FWSEC0292	O177:NM (O177:H25)	*stx*_2c_	River water	2012	Canada	SRR7612106	RRKH00000000	Scaffold	179		78 (Illumina)	4,883,321
FWSEC0293	O157:NM (O157:H7)	*stx*_1a_, *stx*_2c_	River water	2012	Canada	SRR7612096	RRKI00000000	Scaffold	119		72 (Illumina)	5,277,187
FWSEC0294	O26:H11	*stx*_1a_	River water	2012	Canada	SRR7612281	RRKJ00000000	Scaffold	190		93 (Illumina)	5,310,274
FWSEC0296	O111:NM (O111:H8)	*stx*_1a_, *stx*_2a_	River water	2013	Canada	SRR7612341	RRKL00000000	Scaffold	139		124 (Illumina)	5,314,465
FWSEC0297	O111:H8	*stx*_1a_, *stx*_2a_	River water	2013	Canada	SRR7612092	RRKM00000000	Scaffold	161		54 (Illumina)	5,358,475
FWSEC0298	O111:NM (O111:H8)	*stx*_1a_	Water, agricultural (irrigation ditch)	2013	Canada	SRR7612091	RRKN00000000	Scaffold	140		99 (Illumina)	5,289,100
FWSEC0299	O111:H8	*stx*_1a_	Water, agricultural (irrigation ditch)	2013	Canada	SRR7612090	RRKO00000000	Scaffold	140		128 (Illumina)	5,281,401
FWSEC0300	O26:H11	*stx*_1a_	Water, agricultural (irrigation ditch)	2013	Canada	SRR7612557	RRKP00000000	Scaffold	180		112 (Illumina)	5,390,046
FWSEC0301	O26:H11	*stx*_1a_	Water, agricultural (irrigation ditch)	2013	Canada	SRR7612283	RRKQ00000000	Scaffold	192		82 (Illumina)	5,385,968
FWSEC0302	O121:H19	*stx*_2a_	Domesticated livestock, bovine (Bos taurus)	1992	Canada	SRR7612282	RRKR00000000	Scaffold	124		108 (Illumina)	5,029,461
FWSEC0303	O121:H19	*stx*_2a_	Domesticated livestock, bovine (Bos taurus)	1992	Canada	SRR7612533	RRKS00000000	Scaffold	120		75 (Illumina)	5,030,995
FWSEC0304	O121:H19	*stx*_2a_	Clinical, human	1999	Switzerland	SRR7612534	RRKT00000000	Scaffold	108		108 (Illumina)	5,112,326
FWSEC0305	O121:H19	*stx*_2a_	Clinical, human	1999	Switzerland	SRR7612531	RRKU00000000	Scaffold	118		71 (Illumina)	5,167,571
FWSEC0319	O163:H19	*stx*_2a_	Drain water, waste water, culvert	2013	Canada	SRR7612530	RRKW00000000	Scaffold	82		102 (Illumina)	5,024,293
FWSEC0320	O163:H19	*stx*_2a_	Drain water, waste water, culvert	2013	Canada	SRR7612527	RRKX00000000	Scaffold	85		80 (Illumina)	5,121,870
FWSEC0321	O163:NM (O163:H19)	*stx*_2a_	Drain water, waste water, culvert	2013	Canada	SRR7612528	RRKY00000000	Scaffold	207		42 (Illumina)	4,918,527
FWSEC0322	O163:NM (O163:H19)	*stx*_2a_	Drain water, waste water, culvert	2013	Canada	SRR7612535	RRKZ00000000	Scaffold	84		135 (Illumina)	5,019,235
FWSEC0323	O163:H missing (O163:H19)	*stx*_2a_	Drain water, waste water, culvert	2013	Canada	SRR7612536	RRLA00000000	Scaffold	83		94 (Illumina)	5,021,924
FWSEC0324	O163:H19	*stx*_2a_	Drain water, waste water, culvert	2013	Canada	SRR7612172	RRLB00000000	Scaffold	85		106 (Illumina)	5,119,734
FWSEC0325	O163:H19	*stx*_2a_	Drain water, waste water, culvert	2013	Canada	SRR7612171	RRLC00000000	Scaffold	91		109 (Illumina)	5,211,926
FWSEC0327	O163:H19	*stx*_2a_	Drain water, waste water, culvert	2013	Canada	SRR7612173	RRLE00000000	Scaffold	85		108 (Illumina)	5,107,873
FWSEC0329	O163:H19	*stx*_2a_	Drain water, waste water, culvert	2013	Canada	SRR7612167	RRLG00000000	Scaffold	84		110 (Illumina)	5,019,985
FWSEC0330	O163:H19	*stx*_2a_	Drain water, waste water, culvert	2013	Canada	SRR7612170	RRLH00000000	Scaffold	86		96 (Illumina)	5,219,978
FWSEC0332	O54 (O57):H21	*stx*_1d_	River water	2013	Canada	SRR7612180	RRLI00000000	Scaffold	75		93 (Illumina)	4,981,787
FWSEC0333	O54 (O57):H21	*stx*_1d_	River water	2013	Canada	SRR7612537	RRLJ00000000	Scaffold	75		134 (Illumina)	4,920,129
FWSEC0334	O54 (O57):H21	*stx*_1d_	River water	2013	Canada	SRR7612276	RRLK00000000	Scaffold	71		121 (Illumina)	4,982,034
FWSEC0335	O8:H19	*stx*_1a_, *stx*_2d_	Stream water	2013	Canada	SRR7612277	RRLL00000000	Scaffold	95		90 (Illumina)	5,341,011
FWSEC0336	O118:H2	*stx*_1a_	River water	2013	Canada	SRR7612278	RRLM00000000	Scaffold	149		105 (Illumina)	5,256,612
FWSEC0337	O8:H19	*stx*_2a_, *stx*_2d_	River water	2012	Canada	SRR7612279	RRLN00000000	Scaffold	90		111 (Illumina)	5,212,166
FWSEC0338	O168:H8	*stx*_2a_	River water	2012	Canada	SRR7612272	RRLO00000000	Scaffold	111		152 (Illumina)	5,276,982
FWSEC0339	O8:H19	*stx*_2a_, *stx*_2d_	River water	Missing	Canada	SRR7612273	RRLP00000000	Scaffold	88		119 (Illumina)	4,912,080
FWSEC0340	O116:H25	*stx*_2d_	Stream water	2012	Canada	SRR7612274	RRLQ00000000	Scaffold	85		102 (Illumina)	4,957,319
FWSEC0341	O5:NM	*stx*_1a_	Stream water	2012	Canada	SRR7612275	RRLR00000000	Scaffold	143		131 (Illumina)	4,991,314
FWSEC0342	O69:H11	*stx*_1a_	River water	2013	Canada	SRR7612270	RRLS00000000	Scaffold	209		76 (Illumina)	5,350,561
FWSEC0344	O5:NM (O5:H9)	*stx*_1a_	Stream water	2013	Canada	SRR7612401	RRLU00000000	Scaffold	144		99 (Illumina)	4,994,048
FWSEC0346	O22:H8	*stx*_2d_	River water	2013	Canada	SRR7612399	RRLW00000000	Contig	99		102 (Illumina)	4,983,956
FWSEC0347	O174:H21	*stx*_2c_	River water	2013	Canada	SRR7612398	RRLX00000000	Scaffold	102		113 (Illumina)	5,055,323
FWSEC0348	O163:NM (O163:H19)	*stx*_1a_, *stx*_2a_	River water	Missing	Canada	SRR7612405	RRLY00000000	Scaffold	80		126 (Illumina)	5,022,312
FWSEC0350	O163:NM (O163:H19)	*stx*_1a_, *stx*_2a_	Stream water	Missing	Canada	SRR7612403	RRMA00000000	Scaffold	78		165 (Illumina)	5,022,555
FWSEC0351	O163:NM (O163:H19)	*stx*_1a_, *stx*_2a_	River water	Missing	Canada	SRR7612402	RRMB00000000	Scaffold	80		117 (Illumina)	5,021,604
FWSEC0352	O rough:NM (O163:H19)	*stx*_1a_, *stx*_2a_	River water	Missing	Canada	SRR7612397	RRMC00000000	Scaffold	78		105 (Illumina)	5,020,306
FWSEC0353	O163:NM (O163:H19)	*stx*_1a_, *stx*_2a_	Canal water	Missing	Canada	SRR7612396	RRMD00000000	Scaffold	82		124 (Illumina)	5,021,023
FWSEC0354	O rough:NM (O163:H19)	*stx*_1a_, *stx*_2a_	Canal water	Missing	Canada	SRR7612083	RRME00000000	Scaffold	80		121 (Illumina)	5,019,161
FWSEC0355	O163:H19	*stx*_1a_, *stx*_2a_	Canal water	Missing	Canada	SRR7612084	RRMF00000000	Scaffold	76		132 (Illumina)	5,022,505
FWSEC0356	O69:H11	*stx*_1a_	River water	2013	Canada	SRR7612081	RRMG00000000	Scaffold	213		99 (Illumina)	5,417,563
FWSEC0357	O rough:NM (O163:H19)	*stx*_1a_, *stx*_2a_	River water	Missing	Canada	SRR7612082	RRMH00000000	Scaffold	81		153 (Illumina)	5,022,476
FWSEC0358	O rough:NM (O163:H19)	*stx*_1a_, *stx*_2a_	River water	Missing	Canada	SRR7612087	RRMI00000000	Scaffold	77		126 (Illumina)	5,021,430
FWSEC0359	O rough:NM (O163:H19)	*stx*_1a_, *stx*_2a_	River water	Missing	Canada	SRR7612088	RRMJ00000000	Scaffold	87		156 (Illumina)	5,019,056
FWSEC0360	O rough:H21 (O113:H21)	*stx*_2d_	River water	2013	Canada	SRR7612085	RRMK00000000	Scaffold	102		105 (Illumina)	5,129,184
FWSEC0361	O113:H21	*stx*_2d_	River water	2013	Canada	SRR7612086	RRML00000000	Scaffold	136		31 (Illumina)	5,244,379
FWSEC0362	O5:NM (O5:H9)	*stx*_1a_	River water	2013	Canada	SRR7612079	RRMM00000000	Scaffold	146		149 (Illumina)	4,983,571
FWSEC0363	O8:H19	*stx*_1a_, *stx*_2a_	River water	2013	Canada	SRR7612080	RRMN00000000	Scaffold	95		108 (Illumina)	4,909,165
FWSEC0364	O174:H21	*stx*_2a_	River water	2013	Canada	SRR7612237	RRMO00000000	Scaffold	124		88 (Illumina)	5,039,528
FWSEC0365	O128:H2	*stx*_1c_, *stx*_2b_	Stream water	2013	Canada	SRR7612236	RRMP00000000	Scaffold	132		87 (Illumina)	5,412,442
FWSEC0367	O NT:H19 (O8:H19)	*stx*_1a_, *stx*_2a_	Water, agricultural (irrigation ditch)	2013	Canada	SRR7612238	RRMR00000000	Scaffold	83		92 (Illumina)	4,857,754
FWSEC0369	O98:NM (O98:H21)	*stx*_1a_	Water, agricultural (irrigation ditch)	2013	Canada	SRR7612240	RRMT00000000	Scaffold	155		85 (Illumina)	5,326,975
FWSEC0370	O98:NM (O98:H21)	*stx*_1a_	Water, agricultural (irrigation ditch)	2013	Canada	SRR7612243	RRMU00000000	Scaffold	162		96 (Illumina)	5,322,293
FWSEC0371	O157:H7	*stx*_1a_, *stx*_2a_	Water, agricultural (irrigation ditch)	2013	Canada	SRR7612242	RRMV00000000	Scaffold	108		127 (Illumina)	5,209,164
FWSEC0372	O163:H19	*stx*_1a_, *stx*_2a_	River water	Missing	Canada	SRR7612235	RRMW00000000	Scaffold	78		67 (Illumina)	5,022,836
FWSEC0373	O165:H25	*stx*_1a_, *stx*_2a_	Water, agricultural (irrigation ditch)	2013	Canada	SRR7612234	RRMX00000000	Scaffold	163		49 (Illumina)	4,945,961
FWSEC0374	O165:NM (O165:H25)	*stx*_1a_, *stx*_2a_	Water, agricultural (irrigation ditch)	2013	Canada	SRR7612325	RRMY00000000	Scaffold	159		36 (Illumina)	4,948,312
FWSEC0375	O157:H7	*stx*_1a_, *stx*_2a_	Stream water	2013	Canada	SRR7612326	RRMZ00000000	Scaffold	128		238 (Illumina)	5,305,861
FWSEC0376	O88:H25	*stx*_1a_, *stx*_2a_	River water	2013	Canada	SRR7612327	RRNA00000000	Scaffold	84		156 (Illumina)	4,833,021
FWSEC0377	O103:H2	*stx*_1a_	River water	2013	Canada	SRR7612328	RRNB00000000	Scaffold	140		145 (Illumina)	5,304,691
FWSEC0378	O103:H25	*stx*_1a_	Water, agricultural (irrigation ditch)	2013	Canada	SRR7612329	RRNC00000000	Scaffold	165		96 (Illumina)	5,304,528
FWSEC0379	O26:H11	*stx*_1a_	Water, agricultural (irrigation ditch)	2013	Canada	SRR7612330	RRND00000000	Scaffold	178		108 (Illumina)	5,366,964
FWSEC0380	O165:NM (O165:H25)	*stx*_2a_	River water	2013	Canada	SRR7612331	RRNE00000000	Scaffold	162		146 (Illumina)	4,966,985
FWSEC0381	O174:H8	*stx*_1c_, *stx*_2b_	Stream water	2013	Canada	SRR7612332	RRNF00000000	Scaffold	141		129 (Illumina)	5,277,367
FWSEC0382	O128:H2	*stx*_1c_, *stx*_2b_	Stream water	Missing	Canada	SRR7612333	RRNG00000000	Scaffold	143		86 (Illumina)	5,383,076
FWSEC0383	O163:H19	*stx*_1a_, *stx*_2a_	Water, agricultural (irrigation ditch)	2013	Canada	SRR7612334	RRNH00000000	Scaffold	83		103 (Illumina)	5,099,503
FWSEC0384	O128:H2	*stx*_1c_, *stx*_2b_	River water	2013	Canada	SRR7612462	RRNI00000000	Scaffold	164		81 (Illumina)	5,533,994
FWSEC0385	O111:NM (O111:H8)	*stx*_1a_, *stx*_2a_	River water	2013	Canada	SRR7612461	RRNJ00000000	Scaffold	183		88 (Illumina)	5,370,982
FWSEC0386	O8:H9	*stx*_2d_	Water, agricultural (irrigation ditch)	2013	Canada	SRR7612460	RRNK00000000	Scaffold	53		91 (Illumina)	5,789,001
FWSEC0387	O103:H25	*stx*_1a_, *stx*_2a_	Canal water	2013	Canada	SRR7612459	RRNL00000000	Scaffold	164		178 (Illumina)	5,178,285
FWSEC0388	O103:H25	*stx*_1a_	River water	Missing	Canada	SRR7612458	RRNM00000000	Scaffold	155		112 (Illumina)	5,168,250
FWSEC0389	O103:H2	*stx*_1a_	River water	2013	Canada	SRR7612457	RRNN00000000	Scaffold	171		104 (Illumina)	5,301,480
FWSEC0390	O103:H2	*stx*_1a_	River water	2013	Canada	SRR7612456	RRNO00000000	Scaffold	157		73 (Illumina)	5,236,779
FWSEC0391	O103:H2	*stx*_1a_	River water	2013	Canada	SRR7612455	RRNP00000000	Scaffold	182		93 (Illumina)	5,424,966
FWSEC0392	O163:NM (O163:H19)	*stx*_1a_, *stx*_2a_	Domesticated livestock, animal manure, bovine (dairy liquid), dairy farm	1992	Canada	SRR7612454	RRNQ00000000	Scaffold	77		109 (Illumina)	5,022,374
FWSEC0394	O157:H7	*stx*_1a_, *stx*_2a_	Waste water	2010	Canada	SRR7612453	RRNR00000000	Scaffold	113		82 (Illumina)	5,252,251
FWSEC0395	O111:H8	*stx*_1a_	Waste water	2010	Canada	SRR7612155	RRNS00000000	Scaffold	104		97 (Illumina)	5,003,932
FWSEC0397	O174:H21	*stx*_2d_	Waste water	2011	Canada	SRR7612153	RRNU00000000	Scaffold	76		74 (Illumina)	4,911,063
FWSEC0398	O52:H45	*stx*_1c_	Waste water	2011	Canada	SRR7612154	RRNV00000000	Scaffold	100		102 (Illumina)	4,784,323
FWSEC0399	O91:H14	*stx*_1a_	Intake water	2011	Canada	SRR7612151	RRNW00000000	Scaffold	93		102 (Illumina)	5,324,693
FWSEC0400	O113:H4	*stx*_2d_	Intake water	2011	Canada	SRR7612152	RRNX00000000	Contig	128		107 (Illumina)	4,863,421
FWSEC0401	O113:H21	*stx*_2a_	Waste water	2011	Canada	SRR7612149	RRNY00000000	Scaffold	96		118 (Illumina)	5,086,552
FWSEC0402	O171:H2	*stx*_2c_	Intake water	2011	Canada	SRR7612150	RRNZ00000000	Scaffold	111		93 (Illumina)	5,233,546
FWSEC0404	O121:H10	*stx*_2e_	River water	2011	Canada	SRR7612148	RROA00000000	Scaffold	123		71 (Illumina)	5,095,240
FWSEC0405	O157:H7	*stx*_1a_, *stx*_2a_	River water	2011	Canada	SRR7612312	RROB00000000	Scaffold	127		49 (Illumina)	5,340,005
FWSEC0406	O157:H7	*stx*_2c_	Intake water	2011	Canada	SRR7612311	RROC00000000	Scaffold	130		110 (Illumina)	5,239,223
FWSEC0407	O128:NM (O128:H2)	*stx*_1c_, *stx*_2b_	Waste water	2011	Canada	SRR7612314	RROD00000000	Scaffold	132		105 (Illumina)	5,345,000
FWSEC0408	O91:NM (O91:H14)	*stx*_1a_	River water	2012	Canada	SRR7612313	RROE00000000	Scaffold	127		106 (Illumina)	5,346,527
FWSEC0409	O103:H25	*stx*_1a_	River water	2012	Canada	SRR7612308	RROF00000000	Scaffold	159		106 (Illumina)	5,283,169
FWSEC0410	O157:H7	*stx*_1a_, *stx*_2a_	River water	2012	Canada	SRR7612307	RROG00000000	Scaffold	145		110 (Illumina)	5,347,671
FWSEC0411	O121:H19	*stx*_2a_	Stream water	2012	Canada	SRR7612310	RROH00000000	Scaffold	118		123 (Illumina)	5,003,403
FWSEC0413	O157:H7	*stx*_2a_	Waste water	2012	Canada	SRR7612306	RROI00000000	Scaffold	117		118 (Illumina)	5,277,352
FWSEC0414	O153:NM (O153:H2)	*stx*_2f_	Intake water	2012	Canada	SRR7612305	RROJ00000000	Scaffold	167		130 (Illumina)	5,262,960
FWSEC0415	O157:H7	*stx*_2a_	Waste water	2012	Canada	SRR7612551	RROK00000000	Scaffold	125		117 (Illumina)	5,343,022
FWSEC0416	O113:H21	*stx*_2a_	Waste water	2012	Canada	SRR7612550	RROL00000000	Scaffold	98		115 (Illumina)	5,000,817
FWSEC0417	O157:H7	*stx*_1a_	River water	2012	Canada	SRR7612553	RROM00000000	Scaffold	122		129 (Illumina)	5,237,889
FWSEC0418	O139:H19	*stx*_1a_, *stx*_2c_	River water	2012	Canada	SRR7612552	RRON00000000	Scaffold	93		110 (Illumina)	5,051,814
FWSEC0419	O26:H11	*stx*_1a_	Intake water	2012	Canada	SRR7612547	RROO00000000	Scaffold	189		115 (Illumina)	5,475,738
FWSEC0420	O157:H7	*stx*_1a_, *stx*_2a_	River water	2012	Canada	SRR7612546	RROP00000000	Scaffold	111		118 (Illumina)	5,419,843
FWSEC0421	O22:H NT (O22:H8)	*stx*_2f_	River water	2012	Canada	SRR7612526	RROQ00000000	Scaffold	109		112 (Illumina)	5,104,544
FWSEC0422	O103:H2	*stx*_1a_	River water	2013	Canada	SRR7612548	RROR00000000	Scaffold	128		114 (Illumina)	5,192,306
FWSEC0423	O91:NM (O91:H14)	*stx*_1a_	River water	2013	Canada	SRR7612543	RROS00000000	Scaffold	139		112 (Illumina)	5,391,718
FWSEC0424	O182 (O109):H5 (O109:H5)	*stx*_1a_	River water	2013	Canada	SRR7612542	RROT00000000	Scaffold	86		158 (Illumina)	5,195,233
FWSEC0425	O5:NM (O5:H9)	*stx*_1a_, *stx*_2a_	River water	2013	Canada	SRR7612195	RROU00000000	Scaffold	159		123 (Illumina)	5,100,963
FWSEC0426	O128:H2	*stx*_1c_, *stx*_2b_	River water	2013	Canada	SRR7612421	RROV00000000	Scaffold	175		121 (Illumina)	5,573,765
FWSEC0427	O103:H2	*stx*_1a_	River water	2013	Canada	SRR7612418	RROW00000000	Scaffold	145		112 (Illumina)	5,298,328
FWSEC0428	O26:H11	*stx*_1a_	River water	2013	Canada	SRR7612419	RROX00000000	Scaffold	182		137 (Illumina)	5,443,552
FWSEC0429	O1:H20	*stx*_1a_	River water	2013	Canada	SRR7612416	RROY00000000	Scaffold	63		144 (Illumina)	5,071,067
FWSEC0430	O157:H7	*stx*_1a_, *stx*_2a_	River water	2013	Canada	SRR7612417	RROZ00000000	Scaffold	128		88 (Illumina)	5,348,101
FWSEC0431	O103:H11	*stx*_1a_	River water	2013	Canada	SRR7612414	RRPA00000000	Scaffold	183		99 (Illumina)	5,303,799
FWSEC0432	O5:NM (O5:H9)	*stx*_1a_	River water	2013	Canada	SRR7612415	RRPB00000000	Scaffold	159		91 (Illumina)	5,118,324
FWSEC0433	O45:NM (O45:H2)	*stx*_2f_	River water	2013	Canada	SRR7612183	RRPC00000000	Scaffold	147		94 (Illumina)	5,227,140
FWSEC0434	O45:NM (O45:H2)	*stx*_2f_	River water	2013	Canada	SRR7612184	RRPD00000000	Scaffold	152		83 (Illumina)	5,227,905
FWSEC0436	O153:NM (O153:H2)	*stx*_2f_	River water	2013	Canada	SRR7612296	RRPE00000000	Scaffold	158		105 (Illumina)	5,323,485
FWSEC0437	O45:NM (O45:H2)	*stx*_2f_	River water	2013	Canada	SRR7612295	RRPF00000000	Scaffold	156		110 (Illumina)	5,184,594
FWSEC0438	O113:H4	*stx*_2d_	River water	2013	Canada	SRR7612294	RRPG00000000	Contig	122		99 (Illumina)	4,852,452
FWSEC0439	O5:NM (O5:H9)	*stx*_1a_	River water	2013	Canada	SRR7612301	RRPH00000000	Scaffold	164		105 (Illumina)	5,106,616
FWSEC0440	O157:H7	*stx*_1a_, *stx*_2a_	River water	2013	Canada	SRR7612300	RRPI00000000	Scaffold	125		109 (Illumina)	5,285,150
FWSEC0441	O5:NM (O5:H19)	*stx*_1c_	River water	2013	Canada	SRR7612299	RRPJ00000000	Scaffold	120		123 (Illumina)	5,279,452
FWSEC0442	O136:NM (O136:H12)	*stx*_1a_	River water	2013	Canada	SRR7612298	RRPK00000000	Scaffold	211		108 (Illumina)	5,301,182
FWSEC0443	O5:NM (O5:H9)	*stx*_1a_	River water	2013	Canada	SRR7612303	RRPL00000000	Scaffold	165		99 (Illumina)	5,108,519
FWSEC0446	O6:H7 (O6:H11)	*stx*_2a_	Unknown specimen type	2001	Canada	SRR7612142	RRPM00000000	Scaffold	81		76 (Illumina)	4,843,537
FWSEC0447	O26:H11	*stx*_1a_	Unknown specimen type	Not collected	Not collected	SRR7612143	RRPN00000000	Scaffold	197		74 (Illumina)	5,379,480
FWSEC0449	O145:NM (O145:H49)	*stx*_1a_	Unknown specimen type	Not collected	Not collected	SRR7612137	RRPP00000000	Scaffold	137		95 (Illumina)	4,835,826
FWSEC0451	O103:H2	*stx*_1a_	Unknown specimen type	Not collected	Not collected	SRR7612139	RRPQ00000000	Scaffold	129		120 (Illumina)	5,163,904
FWSEC0464	O121:NM (O121:H20)	*stx*_1a_	Unknown specimen type	Not collected	Canada	SRR7612121	RRPZ00000000	Scaffold	62		71 (Illumina)	5,029,443
FWSEC0465	O113:H4 (O113:H20)	*stx*_1a_	Domesticated livestock, bovine, animal manure, agricultural (farm)	Not collected	Canada	SRR7612124	RRQA00000000	Scaffold	61		77 (Illumina)	5,068,214
FWSEC0502	O91:H10	*stx*_2_	Unknown specimen type	Not collected	Not collected	SRR7612438	RRRH00000000	Scaffold	113		62 (Illumina)	5,055,432
FWSEC0508	O26:H11	*stx*_1a_	Unknown specimen type	1984	Canada	SRR7947323	RRRI00000000	Scaffold	202		127 (Illumina)	5,273,275
FWSEC0510	O103:H2	*stx*_1a_	Unknown specimen type	1997	Not collected	SRR7947325	RRRK00000000	Scaffold	166		303 (Illumina)	5,217,653
FWSEC0511	O103:H2	*stx*_1a_	Unknown specimen type	Not collected	Not collected	SRR7947324	RRRL00000000	Scaffold	213		59 (Illumina)	5,271,027
FWSEC0512	O103:H2	*stx*_1a_	Domesticated livestock, bovine, animal manure, agricultural (farm)	1999	Canada	SRR7947327	RRRM00000000	Scaffold	170		303 (Illumina)	5,353,158
FWSEC0514	O103:H2	*stx*_1a_	Domesticated livestock, bovine, animal manure, agricultural (farm)	2004	Canada	SRR7612433	RRRO00000000	Scaffold	141		76 (Illumina)	5,323,892
FWSEC0515	O139:H19	*stx*_1a_, *stx*_2a_	Rectal swab, domesticated livestock, bovine, agricultural (farm)	2012	Canada	SRR7612432	RRRP00000000	Scaffold	74		71 (Illumina)	5,020,809
FWSEC0516	O26:NM (O26:H11)	*stx*_1a_	Rectal swab, domesticated livestock, bovine, agricultural (farm)	2012	Canada	SRR7612435	RRRQ00000000	Scaffold	168		87 (Illumina)	5,327,060
FWSEC0517	O130:H38	*stx*_1a_, *stx*_2a_	Rectal swab, domesticated livestock, bovine, agricultural (farm)	2012	Canada	SRR7612434	RRRR00000000	Scaffold	117		109 (Illumina)	5,052,530
FWSEC0519	O139:H19	*stx*_1a_, *stx*_2a_	Rectal swab, domesticated livestock, bovine, agricultural (farm)	2012	Canada	SRR7947313	RRRS00000000	Scaffold	597		31 (Illumina)	4,805,470
FWSEC0521	O26:NM (O26:H11)	*stx*_1a_	Rectal swab, domesticated livestock, bovine, agricultural (farm)	2012	Canada	SRR7612321	RRRT00000000	Scaffold	178		84 (Illumina)	5,321,164
FWSEC0523	O6:H34	*stx*_1a_, *stx*_2a_	Rectal swab, domesticated livestock, bovine, agricultural (farm)	2012	Canada	SRR7612319	RRRU00000000	Scaffold	92		81 (Illumina)	4,880,556
FWSEC0526	O88:H25	*stx*_1a_, *stx*_2a_	Rectal swab, domesticated livestock, bovine, agricultural (farm)	2012	Canada	SRR7947312	RRRV00000000	Scaffold	599		30 (Illumina)	4,689,586
FWSEC0527	O109:H5	*stx*_1a_	Rectal swab, domesticated livestock, bovine, agricultural (farm)	2012	Canada	SRR7612317	RRRW00000000	Scaffold	74		44 (Illumina)	5,213,197
FWSEC0529	O88:H25	*stx*_2a_	Rectal swab, domesticated livestock, bovine, agricultural (farm)	2012	Canada	SRR7612315	RRRX00000000	Scaffold	95		81 (Illumina)	4,927,775
FWSEC0530	O rough:H38 (O134:H38)	*stx*_2c_	Rectal swab, domesticated livestock, bovine, agricultural (farm)	2012	Canada	SRR7612316	RRRY00000000	Scaffold	69		94 (Illumina)	4,954,025
FWSEC0532	O168:H8	*stx*_2a_	Unknown specimen type	2012	Canada	SRR7947294	RRRZ00000000	Scaffold	383		49 (Illumina)	5,191,428
FWSEC0534	O26:H11	*stx*_1a_	Rectal swab, domesticated livestock, bovine, agricultural (farm)	Not collected	Canada	SRR7947295	RRSA00000000	Scaffold	244		79 (Illumina)	5,309,969
FWSEC0535	O111:H missing (O111:H11)	*stx*_1a_	Rectal swab, domesticated livestock, bovine, agricultural (farm)	Not collected	Canada	SRR7947292	RRSB00000000	Scaffold	317		172 (Illumina)	5,583,793
FWSEC0536	O111:H11	*stx*_1a_	Rectal swab, domesticated livestock, bovine, agricultural (farm)	Not collected	Canada	SRR7947293	RRSC00000000	Scaffold	306		245 (Illumina)	5,754,316
FWSEC0537	O26:H missing (O26:H11)	*stx*_1a_	Rectal swab, domesticated livestock, bovine, agricultural (farm)	Not collected	Canada	SRR7947298	RRSD00000000	Scaffold	203		231 (Illumina)	5,274,187
FWSEC0538	O26:H missing (O26:H11)	*stx*_1a_	Rectal swab, domesticated livestock, bovine, agricultural (farm)	Not collected	Canada	SRR7947299	RRSE00000000	Scaffold	223		187 (Illumina)	5,309,671
FWSEC0539	O111:H missing (O111:H11)	*stx*_1a_	Rectal swab, domesticated livestock, bovine, agricultural (farm)	Not collected	Canada	SRR7947296	RRSF00000000	Scaffold	319		150 (Illumina)	5,736,056
FWSEC0540	O111:H missing (O111:H11)	*stx*_1a_	Rectal swab, domesticated livestock, bovine, agricultural (farm)	Not collected	Canada	SRR7947297	RRSG00000000	Scaffold	318		141 (Illumina)	5,736,086
FWSEC0541	O111:H missing (O111:H11)	*stx*_1a_	Rectal swab, domesticated livestock, bovine, agricultural (farm)	Not collected	Canada	SRR7947286	RRSH00000000	Scaffold	297		154 (Illumina)	5,759,118
FWSEC0542	O111:H missing (O111:H11)	*stx*_1a_	Rectal swab, domesticated livestock, bovine, agricultural (farm)	Not collected	Canada	SRR7947287	RRSI00000000	Scaffold	327		97 (Illumina)	5,716,208
FWSEC0543	O5:H missing (O5:H9)	*stx*_1a_	Rectal swab, domesticated livestock, bovine, agricultural (farm)	Not collected	Canada	SRR7947271	RRSJ00000000	Scaffold	381		56 (Illumina)	5,198,826
FWSEC0544	O111:H missing (O111:H11)	*stx*_1a_	Rectal swab, domesticated livestock, bovine, agricultural (farm)	Not collected	Canada	SRR7947270	RRSK00000000	Scaffold	285		162 (Illumina)	5,698,696
FWSEC0550	O22:H8	*stx*_1a_, *stx*_2a_	Rectal swab, domesticated livestock, bovine, agricultural (farm)	2012	Canada	SRR7612323	RRSL00000000	Scaffold	160		95 (Illumina)	4,924,792
FWSEC0553	O117:H16	*stx*_1a_	Domesticated livestock, bovine, animal manure, agricultural (farm)	Not collected	Canada	SRR7947269	RRSM00000000	Scaffold	102		484 (Illumina)	5,129,595
FWSEC0554	O26:H11	*stx*_1a_	Domesticated livestock, bovine, animal manure, agricultural (farm)	Not collected	Canada	SRR7947268	RRSN00000000	Scaffold	272		59 (Illumina)	5,256,407
FWSEC0555	O22:H8	*stx*_1a_, *stx*_2c_	Domesticated livestock, bovine, animal manure, agricultural (farm)	Not collected	Canada	SRR7612324	RRSO00000000	Scaffold	116		50 (Illumina)	5,150,247
FWSEC0563	O5:NM (O5:H9)	*stx*_1a_	River water	2012	Canada	SRR7612214	RRSU00000000	Scaffold	169		118 (Illumina)	5,346,500
FWSEC0576	O165:NM (O165:H25)	*stx*_1a_, *stx*_2a_	Unknown specimen type	Not collected	Canada	SRR7612216	RRSW00000000	Scaffold	158		145 (Illumina)	4,934,747
FWSEC0595	O26:NM (O26:H11)	*stx*_2a_	Stream water	2012	Canada	SRR7612366	RRSY00000000	Scaffold	90		118 (Illumina)	4,933,762
FWSEC0597	O130:H11 (O130:H38)	*stx*_1a_, *stx*_2a_	Unknown specimen type	2012	Canada	SRR7612365	RRSZ00000000	Scaffold	123		132 (Illumina)	5,047,716
FWSEC0611	O139:H19	*stx*_1a_, *stx*_2a_	Rectal swab, domesticated livestock, bovine, agricultural (farm)	2012	Canada	SRR7612372	RRTF00000000	Scaffold	82		141 (Illumina)	5,012,638
FWSEC0614	O182:NM (O182:H25)	*stx*_2a_	Stream water	2012	Canada	SRR7612501	RRTH00000000	Scaffold	149		103 (Illumina)	5,089,306
FWSEC0620	O6:H34	*stx*_2c_	Rectal swab, domesticated livestock, bovine, agricultural (farm)	2012	Canada	SRR7612497	RRTK00000000	Scaffold	77		109 (Illumina)	4,882,643
FWSEC0621	O136:H12	*stx*_1a_	Unknown specimen type	2012	Canada	SRR7612498	RRTL00000000	Scaffold	196		58 (Illumina)	5,260,253
FWSEC0622	O136:H16	*stx*_1a_	Unknown specimen type	2012	Canada	SRR7612495	RRTM00000000	Scaffold	130		109 (Illumina)	5,284,097
FWSEC0623	O136:H16 (O136:H12)	*stx*_1a_	Unknown specimen type	2012	Canada	SRR7612496	RRTN00000000	Scaffold	195		126 (Illumina)	5,217,336
FWSEC0624	O26:H11	*stx*_1a_	Unknown specimen type	2012	Canada	SRR7612493	RRTO00000000	Scaffold	179		216 (Illumina)	5,286,972
FWSEC0625	O113:H4	*stx*_1a_, *stx*_2d_	Unknown specimen type	2012	Canada	SRR7612494	RRTP00000000	Scaffold	161		81 (Illumina)	4,907,794
FWSEC0626	O139:H19	*stx*_1a_, *stx*_2a_	Rectal swab, domesticated livestock, bovine, agricultural (farm)	2012	Canada	SRR7612134	RRTQ00000000	Scaffold	83		86 (Illumina)	5,015,665
FWSEC0627	O113:NM (O113:H121)	*stx*_2a_	Unknown specimen type	2012	Canada	SRR7612133	RRTR00000000	Scaffold	103		94 (Illumina)	4,992,326
FWSEC0629	O130:H38	*stx*_1a_, *stx*_2a_	Domesticated livestock, farm animal, porcine, animal manure, agricultural (farm)	Not collected	Not collected	SRR7947266	RRTS00000000	Scaffold	264		60 (Illumina)	5,029,373
FWSEC0631	O69:H11	*stx*_1a_	Unknown specimen type	Not collected	Canada	SRR7947264	RRTT00000000	Scaffold	494		50 (Illumina)	5,209,350
FWSEC0632	O111:H missing (O111:H8)	*stx*_1a_, *stx*_2a_	Domesticated livestock, bovine, animal manure, agricultural (farm)	Not collected	Not collected	SRR7947263	RRTU00000000	Scaffold	269		80 (Illumina)	5,455,050

a NT, nontypeable; NM, nonmotile, by traditional lab determination. Isolates were traditionally serotyped at national or provincial reference labs prior to selection for sequencing. Serogroups were then determined algorithmically using EcTyper (https://github.com/phac-nml/ecoli_serotyping) under default settings (min_percentidentity 90; percentLength 50). When traditional and *in silico* serogoup calls varied, both are reported. It is not unusual for genotypically positive strains to be nonmotile under lab determination conditions.

b *stx* allele subtype PCR determination (33) (with algorithmic determination, where noted). Genome assemblies underwent a default BLASTN search (90% threshold for %ID; 60% minimum length) against the Virulence Finder 2.0 Web-based tool (https://cge.cbs.dtu.dk/services/VirulenceFinder/) (34). Reads were mapped using SRST2 (35) and the K-mer analysis toolkit (KAT) sect function (36) to the Flemming Scheutz-curated E. coli virulence database. Although all isolates had been previously determined as genotypically positive for at least one *stx* subtype by PCR, both the A and B subunits of each Stx complex (*stx*_1_ or *stx*_2_) had to be detected during *in silico* screening to be classified as genotypically present.

c WGS, whole-genome sequencing.

d Coding sequences (CDS) for reference genomes FWSEC0001 through FWSEC0011 only.

e A linear sequence in FWSEC0011 that could not be circularized owing to unresolved or collapsed repeats.

### Data availability.

The standardized strain descriptions and accession numbers are presented in Table 1; the genomic data are publicly available in DDBJ/ENA/GenBank under BioProject no. PRJNA287560 and in the Sequence Read Archive under accession no. SRP155537. The versions described are the first versions.
